# Unraveling Biotic and Abiotic Factors Shaping Sugarcane Straw Polyphenolic Richness: A Gateway to Artificial Intelligence-Driven Crop Management

**DOI:** 10.3390/antiox13010047

**Published:** 2023-12-27

**Authors:** Ana L. S. Oliveira, Maria João Carvalho, Poliana Silva, Manuela Pintado, Ana Raquel Madureira

**Affiliations:** CBQF—Centro de Biotecnologia e Química Fina—Laboratório Associado, Escola Superior de Biotecnologia, Universidade Católica Portuguesa, Rua Diogo Botelho 1327, 4169-005 Porto, Portugal; mjcarvalho@ucp.pt (M.J.C.); ppsilva@ucp.pt (P.S.); mpintado@ucp.pt (M.P.); rmadureira@ucp.pt (A.R.M.)

**Keywords:** *Saccharum officinarum*, straw, modeling, polyphenols, biotic and abiotic factors

## Abstract

Sugarcane straw (*Saccharum officinarum*) is a valuable coproduct renowned for its abundant polyphenolic content. However, extracting these polyphenols for natural ingredients faces challenges due to their inherent variability, influenced by biotic stress factors and plant characteristics. We explored the impact of five crucial factors on sugarcane straw polyphenolic diversity: (i) production area (Guariba, Valparaíso), (ii) borer insect (*Diatraea saccharalis*) infestation, (iii) plant age (first to seventh harvest), (iv) harvest season, and (v) plant variety. Response surface methodology (RSM) and artificial neural networks (ANN) were used to optimize polyphenol extraction conditions. A second-order polynomial model guided us to predict ideal sugarcane straw harvesting conditions for polyphenol-rich extracts. The analysis identified CU0618-variety straw, harvested in Guariba during the dry season (October 2020), at the seventh harvest stage, with 13.81% borer insect infection, as the prime source for high hydroxybenzoic acid (1010 µg/g), hydroxycinnamic acid (3119 µg/g), and flavone (573 µg/g) content and consequently high antioxidant capacity. The ANN model surpasses the RSM model, demonstrating superior predictive capabilities with higher coefficients of determination and reduced mean absolute deviations for each polyphenol class. This underscores the potential of artificial neural networks in forecasting and enhancing polyphenol extraction conditions, setting the stage for AI-driven advancements in crop management.

## 1. Introduction

Brazil is a relevant food producer as well as the world’s top producer of sugarcane (*Saccharum* spp.), and the National Supply Corporation—CONAB (2019) reported that during the 2018–2019 harvest, there was about 8.59 million hectares of cultivated land and 620.44 million tons of output. The state of São Paulo was the country’s most significant producer, representing 53.65% of the total processed sugarcane production [1]. Straw residues, often dumped after harvest, are one of the several by-products of sugar production that are generated in large amounts. Those quantities have increased since harvesting transitioned from burning to mechanized [2]. The straw represents a sustainable source of polyphenols, due to the presence of 2,5-dihydroxybenzoic acid, caffeoylquinic acid derivatives, flavones (derivatives of luteolin, apigenin, and tricin), and phenolic acids (sinapic, caffeic, and ferulic acids) [3].

In recent years, the development of novel ingredients that can function as reaction chain breakers and scavengers of damaging free radicals and reactive oxygen species and their use in many applications has led to an expansion of the global polyphenol economy. They have been used as food additives and in brewing goods like liquors and wines (e.g., in baked products, noodles, and pasta) [4,5], as well as in cosmetics to delay the onset of skin aging and enhance moisture and smoothness while reducing roughness and wrinkles [6]. Polyphenols protect against oxidative stress from ultraviolet (UV) irradiation, which is responsible for cutaneous damage and skin cancer [7]. Favorable food safety regulations and increasing public awareness of the health benefits drove the global polyphenol market to 1.6 billion USD in 2020. Projections indicate it will reach 2.7 billion USD by 2030, boasting a 5.2% compound annual growth rate, propelled by expanded usage in the food, beverage, pharmaceutical, and cosmetic industries in the following years [8].

Previously reported findings indicated that phenolic compounds in sugarcane straw extracts are mainly hydroxycinnamic acids, with concentrations reaching approximately 1460.39 µg/g. Chlorogenic acid, neochlorogenic acid, and *p*-coumaric acid dominate this group. Hydroxybenzoic acids form the second-most prevalent class, with concentrations around 727.36 µg/g, while 1-*O*-vanilloyl-β-d-glucose, 2,4-dihydrobenzoic acid, and 3,4-dihydroxybenzaldehyde are the most abundant compounds in this category. Among phenolic compounds, flavones are the least abundant, at 77.56 µg/g. Remarkably, vitexin and isoorientin are the most abundant compounds in this class. These phenolic compounds have been shown to possess potent antioxidant properties, which may contribute to their potential use in various food and pharmaceutical applications [9].

Turning attention to the production of polyphenolic extracts from plant sources, a critical consideration is predicting optimal harvest conditions, especially when dealing with by-products. Plants produce phenolic compounds naturally during growth or as responses to various stimuli, including injuries, infections, or environmental stressors such as heavy metal salts, UV irradiation, and temperature fluctuations [10]. These variables, called abiotic and biotic factors, alter the amount and kind of phenolic chemicals [11,12]. Abiotic factors encompass the nonliving components of the environment, including chemical and physical elements, which influence the behavior of living organisms and the functioning of ecosystems. These factors include soil, water, air, temperature, moisture, and light. In contrast, biotic factors refer to any living organism that affects another organism. Alongside the effects of animals and humans, biotic elements frequently encompass plants, fungi, and microorganisms [10].

In the context of advancing technology, machine learning (ML) and deep learning (DL) within artificial intelligence (AI) have emerged as promising fields with significant potential to enhance various aspects of agricultural practices, including sugarcane crop production and the extraction of valuable by-products such as polyphenols. AI technologies, including artificial neural networks (ANNs), offer valuable tools for collecting and analyzing diverse data in ways that can significantly enhance sugarcane crop production. Recent studies have demonstrated the potential of ML and DL in various aspects of sugarcane production, including crop yield prediction, determination of soil agricultural aptitude, weed identification, and classification of sugarcane varieties. ANNs, in particular, show promise as prognostic tools in modeling studies related to plant cultures, indicating their potential to contribute to the optimization of sugarcane production and the extraction of valuable by-products such as polyphenols [13,14,15,16].

The present study determines how biotic (borer infection, harvestings) and abiotic (geographic zone and season) factors affect polyphenol content (hydroxybenzoic acid, hydroxycinnamic acids, and flavones) in straw from different sugarcane varieties as a potential by-product to produce natural extracts. An estimation was performed with modeling efficiencies of response surface methodology (RSM) and artificial neural networks (ANNs), and they were statistically compared using various parameters, such as coefficient of determination (R^2^) and root mean square error (RMSE).

## 2. Materials and Methods

### 2.1. Sampling Plan

To study the impact of two abiotic factors (geographic zone and season) on sugarcane straw polyphenol richness, the samples were collected during the year 2020 between June and October and from two different geographic areas separated by 274 km (Guariba and Valparaíso, both in São Paulo, Brazil).

**Figure 1:**
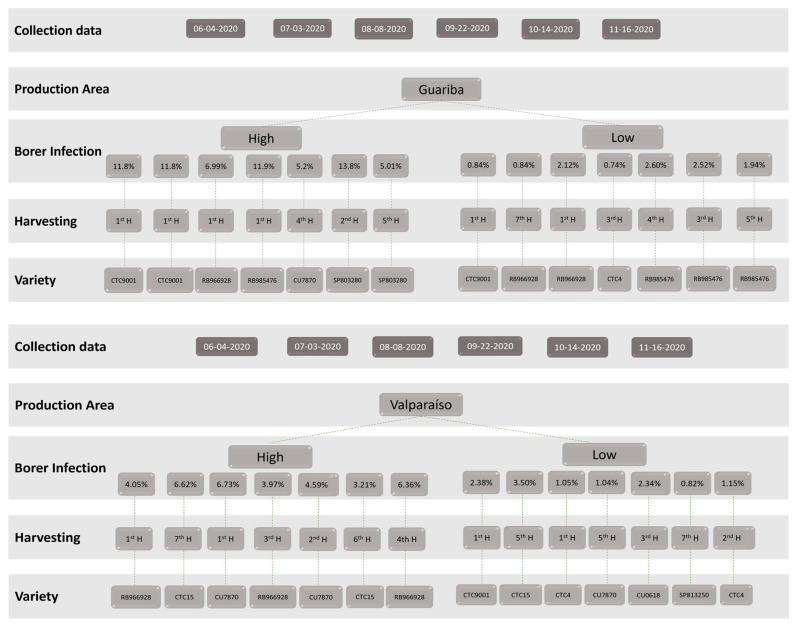
Sampling plan for the sugarcane straw biotic and abiotic effects evaluated in polyphenol content.

At the level of biotic stress, plant samples were selected with a precise level (4–11%) and a low level (0–4%) of borer (*Diatraea saccharalis*) infection and plants from different harvestings (1st–7th). For the study, the sugarcane variety was also considered (SP813250, SP803280, CU0618, RB985476, CTC4, CU7870, CTC15, RB966928, CTC9001). Figure 1 describes the sampling information used in this study.

Samples of 5 kg of straw were systematically collected for each specific combination of variables, including variety, geographic area, season, borer infection level, and plant age (Figure 1). During the sugar extraction harvest, a field worker carefully separated and air-dried the straw before packing it for transport from Brazil to Portugal. The samples were air-shipped at room temperature to the CBQF-UCP laboratory in Porto, Portugal. Subsequently, the material underwent a drying process at 40 °C for 12 h using a ventilated oven (Memmert GmbH + Co.KG, Schwabach, Germany), followed by milling with a grinder (SM100, Retsch, Vila Nova de Gaia, Portugal) to achieve a particle size less than 4 mm. Straw was stored at room temperature and protected from light until beginning assays.

### 2.2. Polyphenolic Extract Production from Sugarcane Straw

In brief, dried sugarcane straw powder was extracted with 50% (*v*:*v*) ethanol in ratio biomass: solvent of 1:10 (*w*:*v*) during 24 h at 30 °C under agitation at 120 rpm (Innova 40 New Brunswick, Eppendorf, Hamburgo, Germany) and protected from light. The solid and liquid fractions were separated by filtration with gauze, and the liquid fraction was centrifuged at 18.671× *g* for 10 min (Sorvall Lynx 4000 centrifuge, Thermo Scientific, Waltham, MA, USA). The ethanol was removed from the liquid fraction evaporation under vacuum with a rotary evaporator at 50 °C, 150 mbar (Heidolph, Walpersdorfer, Germany).

The obtained aqueous fraction was further applied to an Amberlite XAD-2 (Sigma-Aldrich, St. Louis, MO, USA) resin for subsequent purification. The Amberlite XAD-2 was washed with methanol and three times with deionized water. The resin was preconditioned for 12 h in ultrapure water before being used. The resin was used in a ratio of 1:2 (*v*:*w*) and left under agitation of 100 rpm overnight at room temperature. After that, the resin was isolated and washed twice with deionized water at pH 2 to remove any adsorbed sugar. The desorption of the phenolic compounds was performed in two steps, first with a 50% ethanolic solution acidified at pH 2 (HCL, 10 M) under the agitation of 100 rpm at 37 °C overnight and a second desorption in the same conditions for 1 h. The ethanolic extracts were combined and recovered by decantation and filtration (type I filter, V Reis, Lisbon, Portugal). The ethanol was evaporated with a rotary evaporator (50 °C; 150 mbar), and the dried extracts were obtained by freeze-drying (Martin Christ, Osterode am Harz, Germany) for further characterization [3]. Extraction was conducted in triplicate for each sampling condition.

### 2.3. Phenolic Compounds and Organic Acid Analysis by LC-ESI-UHR-QqTOF-MS

The identification and quantification of all phenolic compounds were conducted using liquid chromatography—electrospray ionization—ultrahigh-resolution—quadruple time of flight—mass spectrometry (LC-ESI-UHR-QqTOF-MS) [17]. The dried extracts were first dissolved in a 50% ethanol solution to reach a final concentration of 50 mg/mL and subsequently filtered through a 0.45 µm filter before injection. Separation was carried out on a Bruker Elute series instrument equipped with a UHR-QqTOF mass spectrometer (Impact II, Bruker Daltonics, Bremen, Germany) and a BRHSC18022100 Intensity Solo 2 C18 column (100 × 2.1 mm, 2.2 μm, Bruker, Bremen, Germany).

The separation process involved a flow rate of 0.25 mL/min and followed this elution gradient: 0 min, 0% B; 10 min, 21.0% B; 14 min, 27% B; 18.30 min, 58%; 20.0 min, 100%; 24.0 min, 100%; 24.10 min, 0%; 26.0 min, 0%. The mobile phases used were A (0.1% aqueous formic acid) and B (acetonitrile with 0.1% formic acid).

For the mass spectrometry acquisition, negative ionization mode was employed, with these selected parameters: end-plate offset voltage, 500 V; capillary voltage, 3.0 kV; drying gas temperature, 200 °C; drying gas flow, 8.0 L/min; nebulizing gas pressure, 2 bar; collision radio frequency (RF), ranging from 250 to 1000 Vpp; transfer time, from 25 to 70 μs; collision cell energy, 5 eV. Sodium formate clusters were used for internal mass calibration. Elemental composition was confirmed based on accurate mass and isotope rate calculations designated as mSigma (Bruker Daltonics), and phenolic compounds were identified using their accurate mass [M-H]^−^ using the Bruker Compass DataAnalysis software (version 5.1, Bruker Daltonic GmbH, Bremen, Germany). Quantification results are expressed in micrograms per gram of dry extract.

### 2.4. Antioxidant Activity

The 2.2′-azino-bis (3-ethylbenzothiazoline-6-sulphonic acid) diammonium salt radical cation (ABTS) decolorization experiment was carried out [18] with an ABTS solution prepared by mixing it with K_2_S_2_O_8_ solution at a 1:1 ratio and kept in the dark for 16 h. The solution was diluted with deionized water to achieve an initial OD of 0.700 ± 0.020 at 734 nm. Five sample concentrations were prepared through a 1:1 dilution, starting with a 6.25 mg/mL concentration. Each sample (15 μL, duplicated) was mixed with 200 μL of ABTS, incubated for 5 min at 30 °C in a microplate reader (Synergy H1, Biotek, Winooski, VT, USA), and the OD was measured at 734 nm after incubation.

The DPPH radical cation decolorization assay [18] involved the production of a solution with an OD of 0.600 ± 0.100 at 515 nm by mixing 600 µM of DPPH solution (Sigma-Aldrich, St. Louis, MI, USA) with methanol. Five sample concentrations were prepared through a 1:1 dilution, starting at 6.25 mg/mL. In a microplate, 25 μL of each sample (duplicated) was mixed with 175 μL of DPPH solution, followed by 30 min room-temperature incubation. The OD was then measured at 515 nm using a microplate reader.

For both methodologies, Trolox standard solutions (0.075–0.008 mg/mL) were used for the calibration curve, and the results are expressed as IC50 (mg/mL).

### 2.5. Response Surface Methodology (RSM)

Collection time (date) (X_1_), variety (X_2_), geographical area (X_3_), borer infection level (%) (X_4_), and harvest number (X_5_) were modulated according to a central composite design (CCD). For that, the STATISTICA version 14.0.0.5 (TIBCO Software Inc., Palo Alto, CA, USA) was used. Although the variety variable was not controlled, its significance in influencing polyphenol variability in plants was duly considered for the study. Response variables were estimated using the response surface model described by the following second-order polynomial equation (Equation (1)):

(1) $$Y=\beta_{0}+\beta_{1}X_{1}+\beta_{2}X_{2}+\beta_{3}X_{3}+\beta_{4}X_{1}^{2}+\beta_{5}X_{2}^{2}+\beta_{6}X_{3}^{2}+\beta_{7}X_{1}X_{2}+\beta_{8}X_{1}X_{3}+\beta_{9}X_{2}X_{3}$$

where X_1_, X_2_, X_3_, X_4_, and X_5_ represent the levels of the factors. β_0_–β_9_ represent the coefficient estimates. The variables present in quadratic terms represent the surface curvature, the variables present in linear terms represent the coordinates of the maximum value predicted, and the variables present in bi-factorial cross products represent the axes of the geometric figure formed by partitioning the surface area. The impact of the combinations of the four independent variables on the total phenolic content and phenolic classes’ concentration was examined using the response surface approach.

The optimization of the multi-criteria response surface is based on Derringer’s desirability function. The function converts each variable’s answer into a score of desirability (d) that ranges from 0 (totally unpleasant) to 1 (entirely desirable). The function can be maximized, minimized, or reach a specific goal based on the optimization criterion employed. The desirability function for response variables takes the form of the following equation:

(2) $$d_{i}=0,if\;y_{i}\ll y_{\left(i,min\right)}\;d_{i}={\left[\left(\left(y_{i}-y_{\left(i,min\right)}\right)\right)/\left(\left(y_{\left(i,max\right)}-y_{\left(i,min\right)}\right)\right)\right]}^{\left(w_{i}\right)}\;d_{i}=1,if\;y_{i}\gg y_{\left(i,max\right)}$$

where *y_i_*_,*min*_, and *y_i_*_,_*_max_* are the minimum and maximum desired levels of each response variable *i*, and here the highest and the lowest values of the corresponding quality attribute. Responses below *y_i_*_,*min*_ were assigned a 0 desirability, while responses above *y_i_*_,_*_max_* were assigned a desirability of 1. Between *y_i_*_,*min*_, and *y_i_*_,_*_max_*, the desirability increased linearly by assigning a weight (*w_i_*) of one. A predictive model was used to find the best conditions to obtain the maximum polyphenols in the extracts.

### 2.6. Artificial Neural Networks (ANNs)

STATISTICA version 14.0.0.5 (TIBCO Software Inc.) was used to build and analyze different neural networks to investigate the influence of the input parameters (collection data, production area, borer infection, harvest number, and variety) on the three outputs. From now on, the outputs will be referred to as hydroxybenzoic acids, hydroxycinnamic acids, and flavones.

Data were analyzed using two different types of neural networks: the regression network and the Kohonen network (KN) for categorization (multilayer perceptron, MLP). The experimental dataset was used to generate the RSM model and the ANN models. Of the experimental dataset, 70% (19 points) were used for network training, 15% for validation, and the remaining 15% (4 points) for network testing.

Automated neural networks cluster analysis using the Kohonen training procedure, with the training, testing, and validation data used to build the network. After that, regression neural networks (MLP) were automatically searched for 20 MLP networks, in which all were trained, and five of them were chosen for retention based on their performance throughout training, testing, and validation. The identity, logistic, Tanh, and exponential activation functions were examined for hidden and output neurons. An effective second-order training method was utilized, and the Broyden–Fletcher–Goldfarb–Shanno (BFGS) algorithm was selected. The training algorithm made use of the radial basis function. The activation functions for hidden and output neurons were Gaussian and identity functions. The sum of squares (SOS) was employed as the error function for MLP networks. The Pearson correlation coefficient between experimentally determined values and values predicted by neural networks was measured to evaluate the effectiveness of the proposed neural network models. A global sensitivity analysis was conducted to assess the input variables’ relative significance for the created neural network models. Upon determining the optimal configuration for the ANN method, we conducted a sensitivity analysis to unveil the significance of each operational variable and pinpoint the components crucial for predicting fouling resistance. We applied Equation (3) to achieve this, leveraging the partitioning of connection weights outlined in the Garson equation.

(3) $${RI}_{x}=\frac{\sum_{b=1}^{k_{h}}\left(\left(\frac{{|W}_{xb}|}{\sum_{a=1}^{k_{i}}{|W}_{ab}|}\right)\times |V_{b}|\right)}{\sum_{a=1}^{k_{p}}\left(\sum_{b=1}^{k_{h}}\left(\frac{{|W}_{xb}|}{\sum_{a=1}^{k_{i}}{|W}_{ab}|}\right)\times |V_{b}|\right)}$$

In Equation (3), *RI* denotes the relative importance of the input variable (*x*) regarding the output variable. Here, *k_i_* and *k_h_* refer to the count of input and hidden neurons, while *W_ab_* signifies the connection weights between the input layer and the hidden layer, and *V_b_* represents the connection weight between the hidden layer and the output layer. It is important to clarify that the numerator in Equation (3) signifies the summation of absolute weight products for each input. Conversely, the denominator corresponds to the total of all weights contributing to the hidden unit, considering absolute values.

### 2.7. Comparison of the Prediction Ability of RSM and ANN

The construction of several statistical parameters, including the coefficient of determination (R^2^) and the root mean square error (RMSE), was employed to compare the estimation skills of response surface methodology (RSM) and artificial neural network (ANN) in the context of the study. RSM is a set of statistical methods used for optimizing process variables, and it involves the calculation of summary statistics such as RMSE, adjusted R^2^, and predicted R^2^. The RMSE, which is the square root of the mean square error, is the standard deviation associated with the experimental error. On the other hand, ANN conducts a sensitivity analysis on each model and displays the results in a spreadsheet, which rates the importance of the model’s input variables. The comparison of RSM and ANN in the study involved the assessment of their prediction capacity based on the calculated statistical parameters, providing valuable insights into their respective estimation skills.

## 3. Results and Discussion

### 3.1. Individual Polyphenols Content

The list of individual polyphenols identified among all extracts is given in Table 1, and the quantification of each compound is presented in Table 2 and Table 3.

The analysis of various sugarcane straw extracts revealed the presence of quinic acid esterified with coumaroyl, caffeic, ferulic acid units, and glycosylated protocatechuic acid among the detected metabolites. Hydroxybenzoic acid and dihydroxybenzoic acid linked to sugar moieties were identified, including gentisic acid 5-*O*-β-glucoside. Notably, a metabolite akin to salicylic acid, known as gentisic acid (2,5-dihydrobenzoic acid), plays a crucial role as a signaling molecule in plants’ defense responses against infections [19].

Two types of glycosylation were detected among the flavones, such as C-glucosylated apigenin, luteolin, diosmetin, and *O*-glucosylated tricin. These flavone profiles have been extensively described in different sugarcane by-products, such as juice [20] and leaves [21]. Flavonoid C-glycosides have been shown to have a variety of properties, including antioxidant, insect antifeedant, antibacterial, mycorrhizal symbiosis promoter, and UV-absorbing pigment. These activities need high local concentrations, and many of these chemicals are toxic to plants [22].

Caffeic acid, cis-p-hydroxycinnamic acid, quercetin, apigenin, albanin A, australone A, moracin M, and 5′-geranyl-5,7,2′,4′-tetrahydroxyflavone were the eight phenolic chemicals found in sugarcane’s top ethanolic extracts [23]. Vanillic, ferulic, and syringic acids are among straw’s most prevalent phenolic chemicals [24]. Apigenin diglycoside, named isochaftoside and schaftoside, *p*-coumaric acid, ferulic acid, *p*-hydroxybenzoic acid, caffeic acid, vanillin, protocatechuic acid, and syringic acid were also found in commercial sugarcane juice [25,26]. Our team has reviewed the primary polyphenols found in sugarcane by-products, such as bagasse, juice, leaves, molasses, and rinds [27].

Analyzing the class of polyphenols predominant in all batches for Guariba and Valparaíso, the most representative were hydroxycinnamic acids, especially caffeoylquinic acids derivatives like neochlorogenic acid (maximum of 86 µg/g and 106 µg/g), chlorogenic acid (maximum of 165 µg/g and 276 µg/g), 5-*O*-feruloylquinic acid (maximum of 333 µg/g and 391 µg/g) and coumaric acid derivatives. Within hydroxybenzoic acids, the most representative was 2,5-dihydroxybenzoic acid (maximum of 245 µg/g and 125 µg/g), and finally, in flavones, luteolin-6-C-glucoside (maximum of 290 µg/g and 397 µg/g) (Table 2 and Table 3).

In the natural environment, plants are continuously pressured by biotic and abiotic factors. These adverse circumstances increase reactive oxygen species (ROS) generation, which inhibits plant growth and development and results in significant agricultural output losses [28]. As a defense mechanism against various abiotic stimuli, phenolic accumulation is a characteristic of stressed plants that is often consistent [29]. When plants are exposed to biotic or abiotic stressors, the activation of the phenylpropanoid pathway leads to the production of chlorogenic acid as the primary phenolic compound [30]. With tomato plants subjected to a nematode and water stress simultaneously, a rise in flavonoid and chlorogenic acid levels was also observed [31].

Other plants, such as tea, showed a response to abiotic stressors such as drought, salt, methyl jasmonate, and cold. In this case, the gene expression increased in the phenylpropanoid and lignin pathways and reduced in the flavonoid route. The lignin pathway is crucial for development of plant cell walls, serving as a primary line of defense against environmental stressors. The lignin route is upregulated because of the metabolic flux, where the flavonoid and lignin pathways compete for the same carbon supply. In the presence of abiotic stress and throughout the process of leaf maturation, polyphenols function as the repository for carbohydrates, resulting from photosynthesis in tea plants [32]. In addition, 2,5-dihydroxybenzoic acid was found to be substantially raised in tomato plants infected with the citrus exocortis viroid [33].

With the potential to be used in the food and cosmetic industries, sugarcane straw is a by-product that is a rich source of polyphenols, including 5-*O*-feruloylquinic acid, 2,5-dihydroxybenzoic acid, and luteolin-6-C-glucoside. This makes it a good target for researching the ideal harvesting conditions for high-quality extracts.

The antioxidant potential of sugarcane straw extract powder was assessed using two chemical methods, namely, ABTS and DPPH, with results expressed in Trolox equivalents. As indicated by the ABTS assay, the extract demonstrated the ability to neutralize free radicals at a rate of 0.9–3.6 mg TE/mL, whereas the DPPH assay yielded an antioxidant capacity in a range of 1.0–6.8 mg TE/mL (see Table 4).

Phenolic compounds found in extracts from sugarcane rods have been identified as exhibiting potent antioxidant properties, as indicated by DPPH and FRAP assays. These assays have shown a significant correlation with the levels of phenolic compounds and flavonoids [34]. A comprehensive review summarized the antioxidant potential of various sugarcane products and byproducts, highlighting that the leaves and bagasse contain the highest capacity for neutralizing free radicals, a trait associated with their rich polyphenolic content [35]. Furthermore, another study suggested that sugarcane straw extracts possess antioxidative attributes, which could prove beneficial in mitigating oxidative stress-related diseases or their progression [36]. The current body of evidence points to the utilization of sugarcane juice as a natural source of dietary antioxidants in functional foods, underscoring its exceptional phenolic content, particularly in terms of flavonoids [37].

### 3.2. RSM Modeling

To study how the effect of independent variables like geographic area, borer infection level, harvest number, variety, and harvesting date can influence the recovery of polyphenols from sugarcane straw, a response surface methodology (RSM) analysis was performed. For that, the combination of the selected parameters was determined using central composite design (CCD), and a quadratic model proposed by STATISTICA software was chosen after the analysis of the R^2^ values and *p*-values. This process guarantees that every factor and how it interacts with others is thoroughly investigated. The fitted second-order quadratic model equations’ statistical significance and the importance of each element were evaluated using ANOVA. The adjusted R^2^ in the current investigation was close to the limitations that were deemed acceptable (R^2^ ≥ 0.80), indicating that the experimental data fit the second-order polynomial equations well [38]. Based on the multiple linear regression (MLR) equations, 3D surfaces and contour plots were created to understand the interactions between the independent variables better. The primary and cross-product impacts of the independent variables are more clearly understood thanks to these 3D visualizations that significantly increase on-target replies.

**Figure 2:**
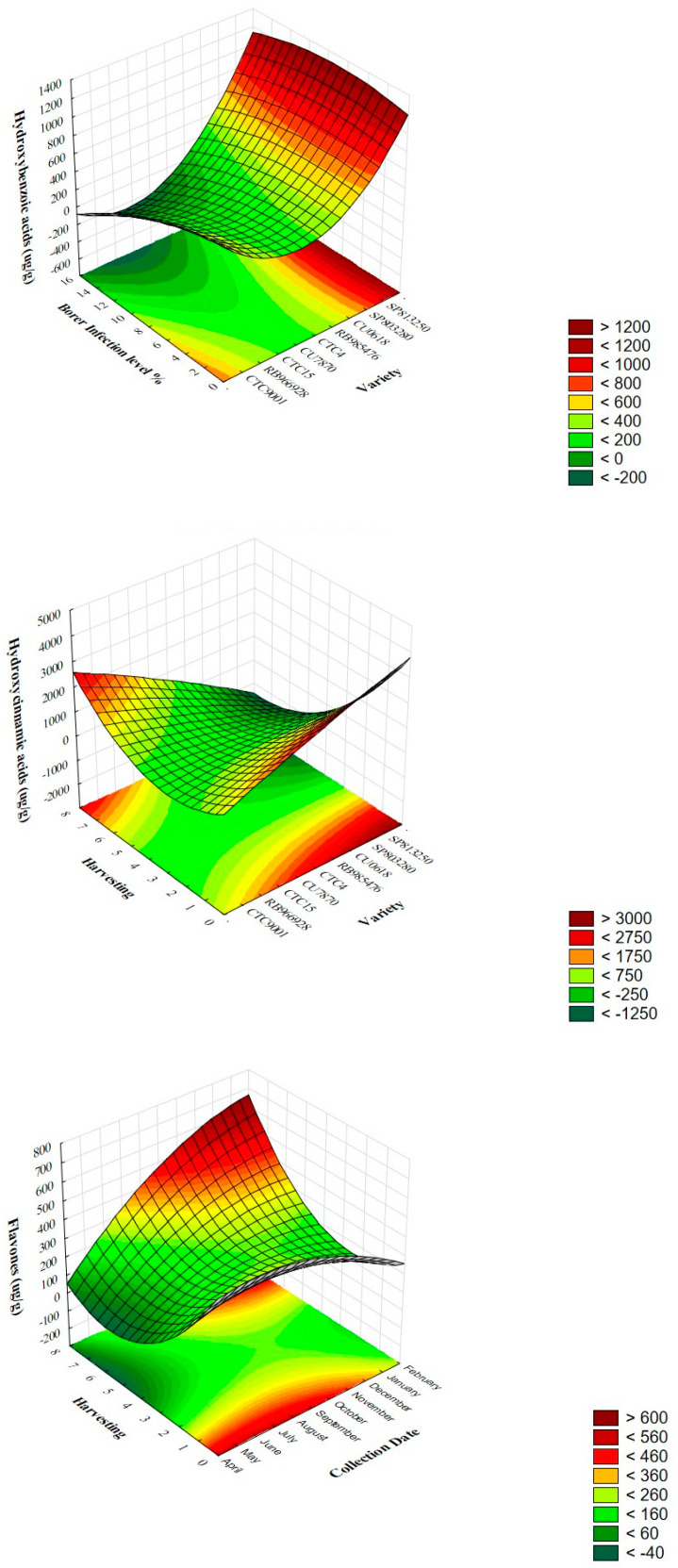
Response surface plot for polyphenol classes (hydroxybenzoic acids, hydroxycinnamic acids, and flavones) quantified in extracts from sugarcane straw harvested under different biotic and abiotic conditions according to the fractional experimental design.

The relationships among the response variables (hydroxybenzoic acid, hydroxycinnamic acid, and flavone content) and the independent variables were evaluated. Based on the analysis of the regression coefficients together with the results of the analyses of variance (ANOVAs) of the second-order polynomial models, the hydroxybenzoic acid class (R^2^ = 0.94) was significantly affected (*p* < 0.05) by the linear term of the variables “variety”, “geographic area”, “infection level” (X_2_, X_3,_ and X_4_), linear and quadratic terms of the variable “harvest number” (X_5_ and X_5_^2^), quadratic term of “variety” (X_2_^2^) and interactive effect between the variables harvesting, variety, geographic area, and collection date (X_1_ X_2_, X_1_ X_3_, X_1_ X_4_, X_2_ X_3_, X_2_ X_5_, and X_3_ X_5_) (Table 5). The linear variables with more potent effects in hydroxybenzoic acid content are represented in 3D surface plots in Figure 2. It was observed that varieties SP803280, SP813250, and CTC9001 were the ones presenting higher content of hydroxybenzoic acids, and the variable “borer infection” had a negative effect, meaning that when the borer infection level increased, the hydroxybenzoic acid content tend to decrease. Through the analysis of the estimated effect for the linear variable (β = –288.93) of “harvest number”, it seems to tend to have more hydroxybenzoic acids in younger plants (1st harvest).

The chosen models for hydroxycinnamic acids and flavones moderately explain the effects of independent variables, since they presented an adjusted R-squared value of 0.71 and 0.78, respectively (Table 5). The lower adjustment for the polynomial model for these two classes could be due to the high variability observed for each compound, since not all presented the same behavior. Due to the redundant effect, the quadratic term for the geographic area was not considered in the polynomial model.

The predictive model for the hydroxycinnamic acids was significantly affected by the linear and quadratic term of “harvest number” (X_5_ and X_5_^2^) and interactions between linear terms for the different variables (X_1_ X_3_, X_2_ X_3_, X_2_ X_4_, X_2_ X_5_, and X_3_ X_5_). Figure 2 represents the combination of variety and harvesting, which had a more substantial effect on hydroxycinnamic acid content. Holder plants (6th–7th harvest) tend to have more of those compounds in combination with varieties like SP813240, SP81340, and CTC9001.

The flavones were significantly affected by the quadratic term “harvest number” (X_5_^2^) and by the interactions between linear terms of “harvesting date”, “harvest number”, “variety”, and “geographic area” (X_1_ X_5_, X_2_ X_5_, and X_3_ X_5_). In this class, the combination of “harvesting” and “collection date” showed that straw from younger plants (1st harvest) harvested between May and August tends to have more flavones (Figure 2).

Antioxidant activity, as assessed using the ABTS method, exhibited significant sensitivity to the linear and quadratic components of “collection time” (X_1_ and X_1_^2^) by the quadratic term of “variety” (X_2_^2^) and the interactions among the linear factors of “harvesting date”, “harvest number”, “variety”, and “geographic area” (X_1_ X_2_, X_1_ X_5_, and X_2_ X_3_). In contrast, the DPPH method did not reveal any notable effects associated with the variables examined.

Within the constraints of the extraction conditions used, this study sought to maximize the extraction of sugarcane straw phenolic compound content. Each projected response was converted using this method into a dimensionless partial desirability function, di, whose values range from 0 for a completely undesired response to 1 for a fully desired response.

For all responses in the current investigation, only one ideal condition was attained: straw from variety CU0618 collected at Guariba near the end of the harvest season (October 2020), using older plants after seven harvestings and with a high level of borer infection (13.81%) (Table 6).

The desirability function D = 1.0 was used to determine the ideal conditions and anticipated values. A positive value for D (>0) denotes that all replies are concurrently in a suitable range. The response numbers are around the goal values because values near 1 suggest that the sum of the various criteria is a global maximum. The predicted values are presented in Table 6, with 977.59 µg/g for hydroxybenzoic acids, 1336.16 µg/g for hydroxycinnamic acid, and 1660.49 µg/g for flavones. For the antioxidant activity, the IC_50_ was 4.84 and 9.96 mg/mL.

### 3.3. Artificial Neural Network (ANN) Modeling

ANNs are a complex optimization and simulation tool with high potential for prediction. According to several published findings, ANN outperforms RSM in terms of its prediction powers [39,40]. As a result, a nonlinear connection between the five input (independent) variables and responses (target outputs) was defined by creating an ANN-based model using a feed-forward back-propagation technique and a topology optimization procedure.

The data from the fifty-six experimental points utilized to create the RSM model were used to train and validate neural networks. Three layers of neurons coupled the feed-forward technique with the multilayer perceptron networks. The first (input) layer of neurons comprised five components, each of which stood for an independent variable. The intermediate (hidden) layer was built to create a model with the least variation between anticipated and experimentally obtained values, and the intermediate (hidden) layer was built. Twenty neurons were present in the intermediate layer of the created ANN model. Three dependent variables were represented by five neurons in the third (output) layer. The model with the highest coefficient of determination (R^2^) and the lowest root mean square error (RMSE) as indicators of the best validation statistics were chosen.

Networks were constructed, and the findings for the best MLP networks (training, test, and validation data), which were chosen based on their performance during the network construction, are shown in Table 7. The names of neural networks indicate how many neurons are present in the input, hidden, and output layers. The gathered results show that in general, the MLP network could recognize and simulate the effect of the input variables on the intended outputs.

**Figure 3:**
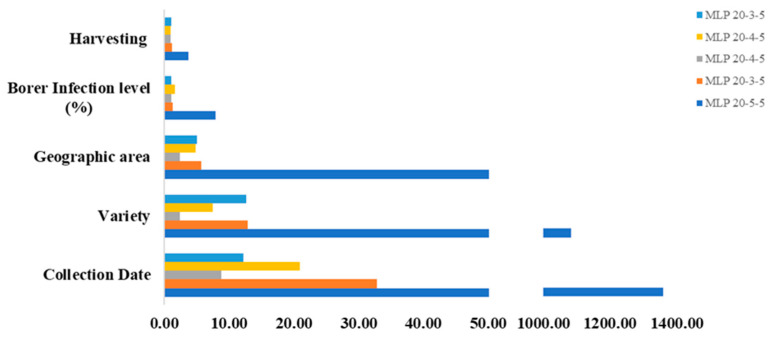
Sensitivity analysis for neural network models that successfully predict phenolic compound classes hydroxybenzoic acids, hydroxycinnamic acids, and flavones and antioxidant activity (ABTS and DPPH) in extracts produced from sugarcane straw considering collection date, variety, geographic area, borer infection level (%), and harvest number.

The input variables that are particularly crucial in the constructed models for the accurate prediction of the desired output variables were found using sensitivity analysis. Sensitivity analysis was carried out for the MLP models for all target outputs. The results are presented in Figure 3 and demonstrate that geographic area, variety, and collection date were the three variables with higher impact on sugarcane straw polyphenol variation for all MPL models.

The validation process employed herein involved the utilization of 15% of the dataset, a choice made due to practical constraints. However, it is essential to acknowledge that this approach may not fully capture the inherent complexities of the entire dataset. Recognizing the imperfections inherent in this validation strategy, leave-one-out cross-validation (LOOCV) would have been a preferable alternative for this specific dataset. LOOCV, by systematically leaving out individual data points during the validation process, offers a more exhaustive and robust evaluation of model performance. While our chosen validation approach was pragmatic, the inclusion of this limitation underscores the need for future investigations to consider employing more comprehensive validation methodologies, such as LOOCV, to further enhance the rigor and generalizability of our findings.

### 3.4. Comparison between RSM and ANN

Here, the prediction performance and estimate skills of the RSM and ANN models were examined. The predicted values of the three target responses (Y_1_, Y_2_, and Y_3_) from the ANN model were statistically evaluated by creating comparative similarity plots. The results show that the ANN model outperformed the RSM model in terms of accuracy, precision, and estimate skills when fitting experimental data to all target answers. The RSM model showed more variance in the residuals, which are the differences between anticipated and actual values, than the ANN model, which showed stable residuals with less change.

Root mean square error (RMSE) and coefficient of determination (R^2^) were also used to compare the RSM and ANN models. Due to its ubiquitous ability to mimic nonlinear systems, ANN has a substantially higher predictive capability than RSM according to the results. In contrast, RSM is only valid for systems with a second-order polynomial regression structure. The RSM requires numerous runs under a standard experimental design for multi-response optimization. However, the ANN can calculate multiple responses in a single run and is independent of the experimental design [41]. To optimize the harvesting conditions of sugarcane to produce the straw extract with a high content of phenolics, the ANN architecture is therefore superior to the RSM model in terms of predictability. It fits the measured responses (hydroxybenzoic acids, hydroxycinnamic acids, and flavones content) (Table 8).

The network MLP 20-5-5 allowed us to get the best fit for all the polyphenolic classes and antioxidant capacity (ABTS and DPPH).

Significant sugarcane losses are a result of biotic stress, and it has been estimated that around 10% of these crop losses are attributable to insect pests, the most significant of which is the sugarcane stem borer (*Diatraea saccharalis* Fabr., *Lepidoptera*, *Crambidae*). The plant’s response to this pest still needs to be fully comprehended. Some authors suggest that the mechanism behind plant protection against insect damage involves the activation of defense proteins. It was reported that sugarcane leaf phenolic extracts show increased proteins after a stimulus with oral secretions from *Diatraea saccharalis* [42,43].

Sugarcane plants may respond to injury by producing secondary compounds, like phenols. A recent study demonstrates a rise in chlorogenic acid and other caffeic acid conjugates produced in the sugarcane leaves of the SP791011 variety in response to herbivory by *Diatraea saccharalis* [44]. Chlorogenic acid is an intermediate in forming insoluble phenolic compounds (e.g., lignin) associated with plant resistance to stress. During herbivory, higher accumulations of chlorogenic acid have been described as an essential defense metabolite in plants [43]. Higher levels of intensity of sugarcane borer infestation can also contribute to the accumulation of phenolic compounds through the action of pathogens that cause red rot. SP80-3280 plants infected with sugarcane borer (19–25%) showed increased phenolic content [45].

Through a process known as ratooning, sugarcane may grow again after being harvested, resulting in repeated harvests of the same crop, usually every 11 to 16 months. During each harvest, sugarcane plants produced less sugar, and this phenomenon was related to management approaches that increased the pressure from pests, diseases, and weeds, reduced soil fertility, compacted the soil, and physically damaged the crop during harvest [46]. We have yet to be aware of any study investigating the influence of the number of sugarcane harvests might have on sugarcane polyphenol content. According to the polynomial model, the “harvest number” variable had a strong influence on the variability of all phenolic classes (Table 6) and the best harvest conditions indicated that after the seventh harvesting (Table 6), the sugarcane straw will contribute to an extract richer in phenolic compounds. Based on the appointed factors associated with “ratooning”, extracts richer in polyphenols should be expected for more harvests since factors like susceptibility to diseases or physically damaged crops tend to produce those secondary metabolites. Mechanically damaged plants create a physical barrier to prevent tissue destruction, including synthesizing polyphenols such as lignin and suberin [43,47].

According to the ANN modeling, the harvest month and variety and geographic area represent the three main variables affecting the phenolic compound content variability (Figure 3).

For meteorological analyses, winter is considered as the quarter of June, July, and August. The winter of 2020 in the capital of São Paulo had rain and above-average temperatures according to measurements carried out by INMET at the meteorological station of Mirante de Santana, in the north of the city of São Paulo. The total rainfall was 198.2 mm, 30% above the historical average. The average maximum temperature was 25.2 °C, and the minimum was 14.7 °C, values that were 1.6 and 1.7 °C, respectively, above the historical average. However, between the months of this study (June–November), when looking for the historical data of precipitation and air temperature, the maximum registered was between October and November with 35 mm and 36.2 °C, respectively. Also, the global radiation was higher in September–December, with a maximum of 3972 Kj/m^2^ in November [48].

**Figure 4:**
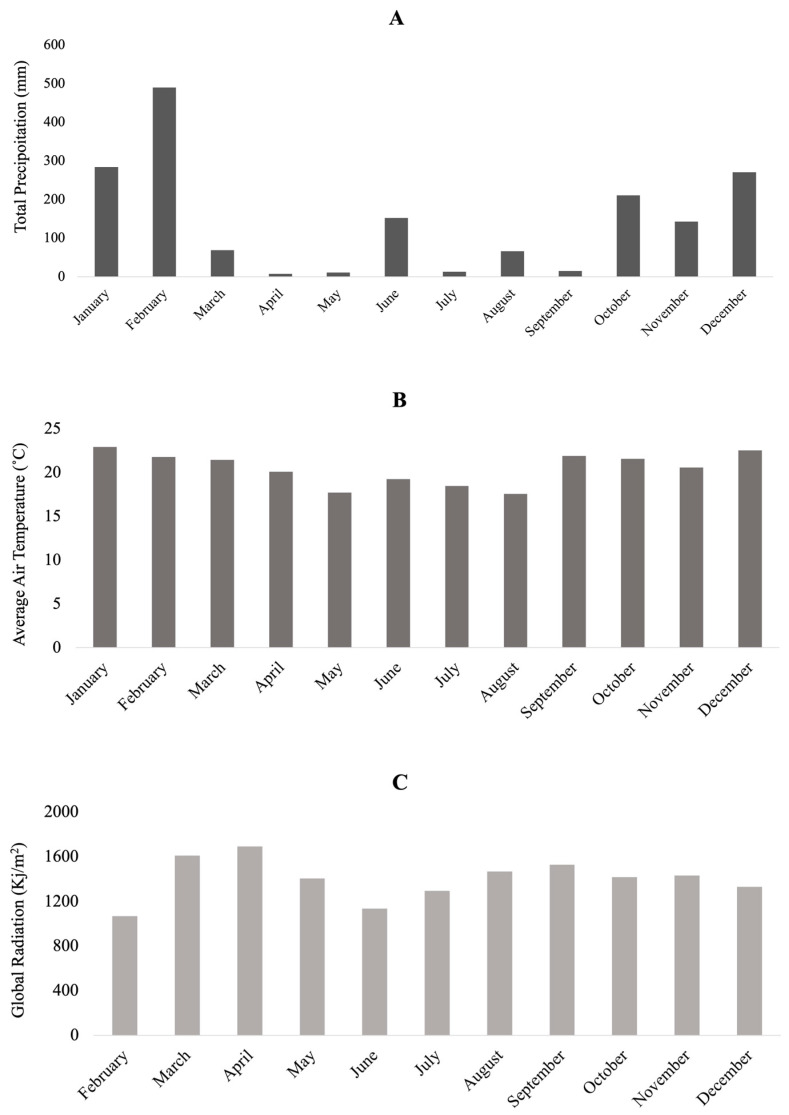
Monthly precipitation (**A**), air temperature (**B**), and global radiation (**C**) from 2020 at the station of Mirante de Santana, São Paulo, Brazil.

In this experiment, the harvesting season was a variable that influenced flavone content, exhibiting higher values when plants were harvested between May and September, a period during which the precipitation was low (Figure 4A). As the polynomial model (Table 6), straw gathered in October was the best month for extracting polyphenols, with the prior months being dry. According to a recent study on sugarcane juice, the crop season (year and season) mainly influenced flavones by lowering rainfall volume in the months before harvest [20]. Activation of the phenylpropanoid biosynthesis pathway in response to drought stress has been observed in several plants, which supports the current study’s findings [29]. Once gathered in the cytoplasm, flavonoids can detoxify H_2_O_2_ molecules produced by drought stress [49].

The maximum amount of global radiation was observed in August–September, and higher average temperatures in September (Figure 4B,C) may also have contributed to the predicted higher polyphenolic content in plants harvested in October according to the meteorological data collected during the study (June–November 2020). Plants produce polyphenols to defend themselves under stress, which can be brought on by radiation, heat, dehydration, etc. As UV-B screens, polyphenols shield the plant from ionizing radiation [5].

In Brazil, there are three main breeding programs run by the Sugarcane Technology Center (CTC varieties), a private company, Instituto Agronômico de Campinas (IAC varieties), supported by the government of São Paulo state, and the Inter-University Network for the Sugar and Ethanol Development (RIDESA—RB varieties), supported by the federal government. In 2015, the variety census for São Paulo state indicated that the four most planted varieties were RB966928 (18%), RB867515 (16%), RB92579 (10%), and RB855156 (7.8%). Two other kinds, RB855453 and SP81-3250, were among the five most cultivated, but not among the five most planted. More resistant types are replacing SP81-3250 because of its vulnerability to orange rust. RB92579 and CTC4 (tenth most cultivated and fifth most planted) might rise in the coming years, given that they were among the five most planted varieties [50].

The varieties analyzed in this study were from SP813250, SP803280, CU0618, RB985476, CTC4, CU7870, CTC15, RB966928, and CTC9001. The polynomial model considered this variety the best for greater phenolic recovery from sugarcane straw (Table 6).

New sugarcane varieties with greater yields are continually being developed and tested to increase the productivity of the Brazilian industry. An appropriate sugarcane variety should be well suited to local changes in temperature, soil type, and cutting technique (manual or mechanical) or ratooning. It should be resistant to pests, infections, and water stress and accumulate high concentrations of sucrose [51].

Plant polyphenol composition is highly dependent on the growing environment, and plants from different geographical regions have demonstrated higher variations in phenolic content due to the different climates and soil compositions [52]. Phenolic compounds and olive leaves calcium, nitrogen, and sodium contents were positively associated. These components have been raised by plants to speed up photosynthetic rate, promote plant development, and boost resistance to drought stress [53]. An example is the olive leaf’s total phenolic level which decreases as geographic altitude decreases. The primary reason for this behavior was attributed to climate variations. Phenolic compounds tend to be less abundant in the leaves of trees grown in windy, humid environments (close to sea level) and more abundant in high-altitude environments with terrestrial and Mediterranean climates, where there are sizable annual temperature variations [11]. The soil nutrient’s influence on plant growth greatly depends on the relationship between water and air in the soil pores. A recent study on different soil media in Hibiscus sabdariffa var. growth and phenolic composition showed no significant influence [54].

In this study, the distance between the two cities was 274 km (Guariba-Valparaiso, São Paulo). The climate at Valparaíso-SP is considered tropical, and soils were classified as sandy loam soil [55]. In Guariba-SP, the soil was classified as clayey according to the Brazilian System of Soil Classification-SIBCS [4]. Valparaíso is characterized as having a higher temperature frequency than Guariba [55].

In wines, for example, it was reported that soil influences vine water status through sandy soils, which have lower water-holding capacity, resulting in wines richer in anthocyanins [56]. Sandy soils are more prone to soil health degradation than clayey soils, and healthier soils were associated with higher sugarcane stalk yields [3]. Based on such presumptions, it should be predicted that plants from Valparaíso will have more polyphenols than plants from Guariba, although other factors associated with the region, such as the climate, may be more important in explaining the polyphenolic difference between them than soil characteristics.

This study presents some limitations, including limited generalizability due to specific conditions in Guariba and Valparaíso and the complexity of models. Findings may not extend to diverse regions or settings with different environmental factors or sugarcane varieties. While optimizing polyphenol extraction, advanced models like RSM and ANN can be resource-intensive and challenging to implement in practice in resource-constrained agricultural settings, potentially limiting their widespread applicability and interpretation.

## 4. Conclusions

In this study, the impact of five biotic and abiotic stresses (geographic area production (Guariba, Valparaíso), level of borer insect (*Diatraea saccharalis*) infection, harvest number (first to seventh), harvest season and plant variety) were evaluated in sugarcane straw as a potential by-product for natural extract production richer in polyphenols. The ideal extraction settings were found using a response surface methodology (RSM) based on a central composite design to optimize the yield of all target compounds concurrently. The optimal conditions were plants from the CU0618 variety collected at Guariba in the dry season (October 2020), using holder plants (seventh harvest), with a level of borer infection of 13.81%. The three most significant factors for the richness of sugarcane straw polyphenols, according to the artificial neural network (ANN) model, are season, region, and variety.

Compared to RSM models, the ANN model had better coefficients of determination values, indicating a superior potential for prediction. The study’s findings that are being presented advance our understanding of the extraction of essential compounds needed for further development, separation, purification, and scale-up processes at the industrial level.

In practice, the developed model aid in monitoring year-round biotic and abiotic conditions, offering predictive insights for strategic straw usage in extract production. These findings highlight the potential for utilizing sugarcane straw as a source of valuable polyphenols, contributing to the development of sustainable practices in the sugar production industry.

## Figures and Tables

**Table 1:** Phenolic compounds identified in sugarcane straw extracts.

Compound Name	Formula	[M-H]^−^	Fragments MS^2^
Hydroxybenzoic acids			
1-*O*-Vanilloyl-β-d-glucose	C_14_H_18_O_9_	329	167
Vanillic acid	C_8_H_8_O_4_	167.0350	108, 119, 152
Protocatechuic acid	C_7_H_6_O_4_	153.0193	109, 153
2,5-Dihydrobenzoic acid	C_7_H_6_O_4_	153.0193	109, 153
Gentisic acid 2-*O*-β-glucoside	C_13_H_15_O_9_	315.0709	108, 152
Gentisic acid 5-*O*-β-glucoside	C_13_H_15_O_9_	315.0709	109, 153
Protocatechuic acid 4-β-glucoside	C_13_H_15_O_9_	315.0709	109, 153
4-Hydroxybenzoic acid	C_7_H_5_O_3_	137.0221	137
3,4-Dihydroxybenzaldehyde	C_7_H_5_O_3_	137.0221	93, 137
4-Hydroxybenzaldehyde	C_7_H_5_O_2_	121.0276	121
Hydroxybenzoic-4-β-glucoside	C_13_H_15_O_8_	299.0717	137
Hydroxycinnamic acids			
Neochlorogenic acid	C_16_H_18_O_9_	353.08781	135, 179, 191
Chlorogenic acid	C_16_H_18_O_9_	353.08781	191
4-Caffeoylquinic acid	C_16_H_18_O_9_	353.08781	135, 173, 179, 191
*cis*-5-*O*-p-Coumaroylquinic acid	C_16_H_18_O_8_	337.09289	93, 163, 173, 191
5-*O*-Feruloylquinic acid	C_17_H_20_O_9_	367.0971	134, 193
*trans*-3-Feruloylquinic acid	C_17_H_19_O_9_	367.0596	173
Feruloylquinic acid isomers	C_17_H_20_O_9_	367.0961	173, 191
Caffeic acid	C_9_H_8_O_4_	179.0317	135, 179
Ferulic acid derivatives	C_10_H_10_O_4_	193.05063	134, 191
Coumaric acid derivatives	C_9_H_8_O_3_	163.04007	119, 163
Caffeoylquinic acid	C_16_H_18_O_9_	515.11	515
4,5-Dicaffeoylquinic acid	C_25_H_24_O_12_	515.11	173, 179, 191, 353
Caffeoylshikimic acid	C_16_H_16_O_8_	335.071	135, 161, 179
Flavones			
Apigenin-8-C-glucoside	C_21_H_20_O_10_	431.09837	311, 341, 431
Isovitexin 2″-*O*-arabinoside	C_26_H_27_O_14_	563.14063	353, 443
Isoschaftoside	C_26_H_27_O_14_	563.14063	353, 473
Neoschaftoside	C_26_H_27_O_14_	563.14063	399, 473
Apigenin-6-C-glucosyl-8-C-arabinoside	C_26_H_28_O_14_	563.14063	353, 443
Luteolin-6-C-glucoside	C_21_H_20_O_11_	447.09329	327, 357
Luteolin-8-C-glucoside	C_21_H_20_O_11_	447.09329	327, 357
Apigenin 7-*O*-neohesperidoside	C2_7_H_29_O_14_	577.1563	293, 413
Luteolin	C_15_H_10_O_6_	285.04046	285
6-Methoxyluteolin 7-rhamnoside	C2_2_H_21_O_11_	461.1089	461
Diosmetin	C_16_H_12_O_6_	299.0502	284, 299
Tricin-*O*-neohesperoside isomer	C_29_H_33_O_16_	637.1638	329
Tricin-7-*O*-glucoside	C_25_H_31_O_10_	491.1826	329
Tricin-7-*O*-rhamnosyl-glucuronide	C_36_H_27_O_12_	651.144	329
Tricin-4-(*O*-erythro) ether glucoside	C_33_H_35_O_16_	687.1786	195, 329, 491, 525
Tricin	C_17_H_13_O_7_	329.0667	299

**Table 2:** (**a**) Polyphenols identified with concentrations > 20 µg/g extract in sugarcane straw extracts of plants collected in the Guariba area with borer infection (BHI) and low infection (BLI) between June and August 2020 from different harvests (1st–7th). Values represent the average ± standard deviation. (**b**) Polyphenols identified with concentrations > 20 µg/g extract in sugarcane straw extracts of plants collected in the Guariba area with borer infection (BHI) and low infection (BLI) between September and November 2020 from different harvests (1st–7th). Values represent the average ± standard deviation.

**(a)**								
Identified Compound	4 June 2020		3 July 2020		24 July 2020		8 August 2020	
	CTC9001		CTC9001	RB966928	RB966928		RB985476	CTC4
	BHI	BLI	BHI	BLI	BHI	BLI	BHI	BLI
	1st H	1st H	1st H	7th H	1stH	1st H	1st H	3rd H
Hydroxybenzoic acids	µg/g DW extract							
1-*O*-Vanilloyl-β-d-glucose	-	-	-	-	-	-	301.1 ± 31.9	122.1 ± 78.3
Vanillic acid	-	-	-	-	-	-	72.9 ± 9.9	40.7 ± 19.2
2,5-Dihydrobenzoic acid	38.8 ± 0.4	122.0 ± 4.6	100.8 ± 8.7	43.5 ± 6.8	245.5 ± 17.3	171.3 ± 1.0	156.7 ± 5.5	135.4 ± 6.2
Gentisic acid 2-*O-*β-glucoside	-	-	4.6 ± 0.2	3.9 ± 0.7	32.2 ± 1.0	46.3 ± 3.8	32.3 ± 5.1	19.8 ± 0.1
Gentisic acid 5-*O*-β-glucoside	1.4 ± 0.1	3.2 ± 0.2	5.3 ± 0.3	3.8 ± 0.1	30.2 ± 0.8	67.3 ± 7.1	26.1 ± 2.2	14.3 ± 0.6
4-Hydroxybenzoic acid	0.2 ± 0.0	1.8 ± 0.1	7.7 ± 2.5	17.1 ± 0.9	48.5 ± 1.5	37.6 ± 4.8	28.2 ± 2.7	32.7 ± 0.2
4-Hydroxybenzaldehyde	16.4 ± 0.8	16.3 ± 0.8	36.9 ± 1.0	20.1 ± 1.9	20.8 ± 1.0	26.8 ± 1.0	22.6 ± 1.5	37.3 ± 2.2
Hydroxybenzoic-4-β-glucoside	-	-	-	-	9.4 ± 0.5	26.6 ± 0.9	5.0 ± 0.1	5.8 ± 0.3
Hydroxycinnamic acids	µg/g DW extract							
Neochlorogenic acid	0.8 ± 0.2	2.2 ± 0.1	1.4 ± 0.5	0.2 ± 0.0	52.3 ± 0.6	22.5 ± 0.3	55.1 ± 6.4	62.8 ± 1.0
Chlorogenic acid	1.9 ± 0.2	3.6 ± 0.1	4.8 ± 0.7	3.5 ± 1.9	165.7 ± 19.1	85.4 ± 0.5	106.0 ± 8.4	131.5 ± 4.1
4-Caffeoylquinic acid	1.2 ± 0.1	2.2 ± 0.1	0.8 ± 0.4	0.2 ± 0.0	26.5 ± 0.8	15.1 ± 0.5	28.0 ± 2.5	34.4 ± 1.4
5-*O*-Feruloylquinic acid	14.8 ± 1.3	54.5 ± 1.7	124.4 ± 10.3	18.2 ± 5.8	333.5 ± 26.9	200.1 ± 2.6	186.2 ± 6.9	240.3 ± 11.4
*trans*-3-Feruloylquinic acid	-	-	88.8 ± 4.4	17.9 ± 1.9	44.3 ± 1.7	27.1 ± 0.3	87.4 ± 5.8	121.9 ± 2.5
Feruloylquinic acid isomer	9.2 ± 1.2	25.1 ± 0.1	-	-	169.5 ± 11.7	108.7 ± 1.7	11.7 ± 0.7	12.2 ± 0.2
Caffeic acid	1.7 ± 0.2	3.3 ± 0.1	8.1 ± 0.5	2.1 ± 1.7	17.8 ± 1.1	12.8 ± 0.2	11.5 ± 0.3	13.9 ± 1.4
Ferulic acid	14.3 ± 0.2	33.0 ± 0.1	42.2 ± 1.3	28.4 ± 0.8	57.9 ± 1.6	47.7 ± 3.8	40.6 ± 0.7	48.5 ± 0.7
*p*-Coumaric acid	63.9 ± 5.6	59.7 ± 0.9	120.6 ± 11.9	102.4 ± 17.3	63.8 ± 1.4	59.7 ± 11.0	74.2 ± 1.6	83.0 ± 2.5
Caffeoylquinic acid	3.2 ± 0.4	13.7 ± 0.6	24.1 ± 2.1	22.5 ± 5.0	38.3 ± 0.4	26.9 ± 0.8	37.2 ± 1.8	46.3 ± 3.2
Flavones	µg/g DW extract							
Apigenin-8-C-glucoside	13.5 ± 0.5	32.9 ± 1.1	38.6 ± 1.0	27.8 ± 0.9	127.9 ± 10.0	70.6 ± 5.0	72.2 ± 1.5	80.0 ± 1.6
Isovitexin-2″-O-arabinoside	1.20 ± 0.1	4.3 ± 0.1	3.5 ± 0.2	3.7 ± 1.1	20.1 ± 1.9	27.7 ± 0.7	3.5 ± 0.3	2.0 ± 0.1
Isoschaftoside	17.07 ± 1.4	40.7 ± 2.6	44.1 ± 4.5	38.0 ± 7.0	4.7 ± 0.6	6.73 ± 0.1	55.4 ± 3.3	37.2 ± 1.1
Luteolin-6-C-glucoside	-	-	101.6 ± 6.5	56.9 ± 17.2	250.3 ± 42.1	221.0 ± 0.9	215.4 ± 20.9	290.1 ± 1.0
Luteolin-8-C-glucoside	-	-	-	-	42.9 ± 4.4	9.7 ± 0.5	22.3 ± 2.1	29.2 ± 1.8
Apigenin-7-*O*-neohesperidoside	4.1 ± 0.6	9.0 ± 0.3	2.6 ± 0.4	5.64 ± 1.2	28.9 ± 1.5	17.6 ± 1.1	14.3 ± 0.9	21.9 ± 0.2
Tricin	3.8 ± 0.1	7.3 ± 0.6	0.01 ± 0.0	0.01 ± 0.0	8.7 ± 0.5	9.7 ± 0.4	14.4 ± 1.0	14.3 ± 0.9
**(b)**								
Identified Compound	22 September 2020				14 October 2020		16 November 2020	
	CU7870		RB985476		SP803280		SP803280	
	BHI		BLI		BHI	BLI	BHI	BLI
	4th H		4th H		2nd H	3rd H	5th H	5th H
Hydroxybenzoic acids	µg/g DW extract							
1-*O*-Vanilloyl-β-d-glucose	158.6 ± 14.0		60.1 ± 60.0		123.4 ± 1.3	126.4 ± 70.0	-	-
Vanillic acid	51.9 ± 2.9		53.3 ± 0.3		52.0 ± 1.7	53.7 ± 19.0	38.4 ± 4.9	43.3 ± 7.3
2,5-Dihydrobenzoic acid	106.3 ± 25.5		102.9 ± 1.7		170.5 ± 5.8	155.8 ± 7.4	14.5 ± 0.6	16.1 ± 0.3
Gentisic acid 2-*O*-β-glucoside	19.0 ± 3.0		20.9 ± 1.6		27.4 ± 1.4	22.7 ± 0.2	0.4 ± 0.2	0.3 ± 0.3
Gentisic acid 5-*O*-β-glucoside	18.9 ± 1.9		20.6 ± 1.7		21.8 ± 1.2	17.6 ± 0.2	0.5 ± 0.1	0.3 ± 0.1
4-Hydroxybenzoic acid	33.6 ± 0.5		30.3 ± 2.6		45.7 ± 1.8	73.6 ± 3.1	8.7 ± 1.7	5.7 ± 0.6
4-Hydroxybenzaldehyde	12.1 ± 0.9		9.6 ± 0.1		48.9 ± 1.4	61.4 ± 4.3	5.9 ± 0.6	4.3 ± 0.4
Hydroxybenzoic-4-β-glucoside	4.2 ± 1.3		5.3 ± 0.9		3.9 ± 0.2	2.9 ± 0.1	-	-
Hydroxycinnamic acids	µg/g DW extract							
Neochlorogenic acid	83.1 ± 3.2		59.6 ± 3.3		86.0 ± 3.7	81.1 ± 0.5	-	-
Chlorogenic acid	103.7 ± 15.7		100.0 ± 5.3		70.7 ± 3.8	71.5 ± 0.5	2.6 ± 0.1	2.5 ± 0.3
4-Caffeoylquinic acid	43.4 ± 3.2		40.4 ± 1.5		42.9 ± 1.1	44.7 ± 1.5	-	-
5-*O*-Feruloylquinic acid	102.4 ± 22.1		122.2 ± 2.5		163.9 ± 0.2	153.7 ± 2.8	6.2 ± 0.1	8.1 ± 0.4
*trans*-3-Feruloylquinic acid	11.8 ± 1.0		9.7 ± 0.6		94.5 ± 0.7	57.8 ± 25.9	3.5 ± 0.2	4.5 ± 0.2
Feruloylquinic acid isomer	79.8 ± 1.0		74.5 ± 2.7		9.3 ± 0.6	49.4 ± 10.0	-	-
Caffeic acid	35.8 ± 8.9		42.6 ± 0.7		24.2 ± 1.4	46.1 ± 3.4	5.8 ± 0.3	6.5 ± 0.5
Ferulic acid	40.3 ± 10.0		50.2 ± 0.6		31.1 ± 0.8	34.3 ± 0.6	6.7 ± 1.0	7.0 ± 1.1
*p*-Coumaric acid	30.8 ± 1.7		38.5 ± 1.1		37.4 ± 2.2	53.6 ± 1.6	21.4 ± 0.7	22.0 ± 1.3
Caffeoylquinic acid	15.5 ± 2.2		17.4 ± 0.2		27.6 ± 2.8	26.4 ± 2.8	6.5 ± 0.4	8.6 ± 0.8
Flavones	µg/g DW extract							
Apigenin-8-C-glucoside	59.8 ± 2.5		56.6 ± 6.7		64.7 ± 3.5	52.7 ± 1.3	11.4 ± 1.0	12.0 ± 0.5
Isovitexin 2″-*O*-arabinoside	1.3 ± 0.1		1.5 ± 0.1		-	-	-	-
Isoschaftoside	22.3 ± 1.5		24.4 ± 0.5		15.1 ± 2.0	17.7 ± 0.2	7.3 ± 0.8	8.4 ± 0.4
Luteolin-6-C-glucoside	129.8 ± 17.5		137.5 ± 2.9		174.5 ± 22.7	168.6 ± 1.0	37.8 ± 4.4	40.3 ± 1.5
Luteolin-8-C-glucoside	13.9 ± 1.4		3.4 ± 0.1		7.8 ± 1.4	6.6 ± 0.4	12.4 ± 0.4	12.8 ± 1.6
Apigenin 7-*O*-neohesperidoside	27.0 ± 3.3		22.6 ± 0.2		11.3 ± 0.5	5.5 ± 0.2	-	-
Tricin	33.1 ± 1.1		27.9 ± 1.0		9.7 ± 0.5	9.9 ± 0.6	4.8 ± 0.4	5.1 ± 0.5

**Table 3:** (**a**) Polyphenols identified with concentrations > 20 µg/g extract in sugarcane straw extracts from plants collected in the Valparaiso area with borer high-infection (UHI) and low-infection (ULI) levels between June and August 2020 from different harvests (1st–7th). Values represent the average ± standard deviation. (**b**) Polyphenols identified with concentrations > 20 µg/g extract in sugarcane straw extracts from plants collected in the Valparaiso area with borer high-infection (UHI) and low-infection (ULI) levels between September and November 2020 from different harvests (1st–7th). Values represent the average ± standard deviation.

**(a)**								
Identified Compound	4 June 2020		3 July 2020		24 July 2020		8 August 2020	
	RB966928	CTC9001	CTC15		CU7870	CTC4	RB966928	CU7870
	UHI	ULI	UHI	ULI	UHI	ULI	UHI	ULI
	1st H	1st H	7th H	5th H	2nd H	1st H	3rd H	4th H
Hydroxybenzoic acids	µg/g DW extract							
1-*O*-Vanilloyl-β-d-glucose	-	-	-	-	-	-	36.2 ± 0.4	21.3 ± 10.6
Vanillic acid	-	-	-	-	-	-	21.2 ± 3.5	11.7 ± 1.8
2,5-Dihydrobenzoic acid	16.0 ± 0.2	18.1 ± 0.5	142.4 ± 2.3	62.6 ± 1.8	125.7 ± 13.0	145.8 ± 12.1	17.6 ± 3.2	10.5 ± 1.2
Gentisic acid 2-*O*-β-glucoside	-	-	19.3 ± 0.5	11.2 ± 0.2	20.8 ± 1.7	24.8 ± 1.3	-	-
Gentisic acid 5-*O*-β-glucoside	-	-	14.2 ± 0.1	6.9 ± 0.3	14.5 ± 0.7	20.4 ± 0.0	-	-
4-Hydroxybenzoic acid	1.4 ± 0.5	0.5 ± 0.1	43.6 ± 1.6	22.2 ± 1.1	77.8 ± 0.5	43.8 ± 1.0	3.0 ± 0.4	1.7 ± 1.7
3,4-Dihydroxybenzaldehyde	1.8 ± 0.0	3.9 ± 1.0	5.1 ± 0.2	2.3 ± 0.6	2.7 ± 0.2	18.3 ± 0.5	-	-
4-Hydroxybenzaldehyde	11.2 ± 0.8	29.8 ± 0.7	19.2 ± 0.1	35.3 ± 1.1	17.8 ± 1.0	38.2 ± 1.1	24.9 ± 0.1	22.6 ± 0.6
Hydroxycinnamic acids	µg/g DW extract							
Neochlorogenic acid	1.0 ± 0.2	1.1 ± 0.3	26.4 ± 1.5	7.2 ± 1.2	103.5 ± 12.0	106.7 ± 9.0	-	-
Chlorogenic acid	2.0 ± 0.2	1.1 ± 0.3	68.9 ± 3.2	21.6 ± 3.3	255.1 ± 4.4	272.8 ± 6.0	2.5 ± 0.7	2.4 ± 0.1
4-Caffeoylquinic acid	1.3 ± 0.1	1.2 ± 0.1	14.1 ± 1.1	5.9 ± 0.8	60.3 ± 3.0	51.3 ± 2.6	-	-
5-*O*-Feruloylquinic acid	8.2 ± 1.3	13.5 ± 1.0	263.2 ± 1.3	105.3 ± 2.9	211.5 ± 20.0	391.5 ± 8.1	4.5 ± 1.5	2.0 ± 0.2
trans-3-Feruloylquinic acid	-	-	16.8 ± 0.4	7.6 ± 0.4	12.9 ± 1.9	65.5 ± 4.7	4.7 ± 0.6	2.4 ± 0.0
Feruloylquinic acid isomer	6.0 ± 1.1	10.6 ± 1.0	118.5 ± 1.3	86.8 ± 0.5	100.4 ± 9.9	204.1 ± 12.6	-	-
Caffeic acid	1.9 ± 0.2	1.4 ± 0.3	14.1 ± 0.7	5.6 ± 0.2	53.6 ± 12.0	25.8 ± 1.4	3.3 ± 0.7	3.7 ± 0.3
Ferulic acid	14.2 ± 1.0	13.2 ± 0.9	52.5 ± 0.5	46.4 ± 0.1	40.4 ± 1.7	56.8 ± 2.2	17.1 ± 0.6	16.2 ± 0.4
*p*-Coumaric acid	71.3 ± 5.6	100.8 ± 5.8	75.6 ± 1.1	125.3 ± 11.5	59.8 ± 1.7	76.6 ± 2.6	95.9 ± 5.6	106.3 ± 2.7
Caffeoylquinic acid	2.7 ± 0.4	2.8 ± 0.3	49.9 ± 1.8	53.7 ± 2.5	43.3 ± 5.9	58.9 ± 0.4	4.9 ± 0.7	3.1 ± 0.4
4,5-Dicaffeoylquinic acid	-	-	-	-	41.7 ± 2.9	11.2 ± 0.6	-	-
Flavones	µg/g DW extract							
Apigenin-8-C-glucoside	9.1 ± 0.2	7.0 ± 0.3	93.2 ± 0.6	35.5 ± 1.5	103.8 ± 2.7	124.2 ± 2.0	11.2 ± 0.3	8.8 ± 0.2
Isovitexin 2″-*O*-arabinoside	0.8 ± 0.1	0.4 ± 0.0	7.0 ± 0.2	6.9 ± 0.9	51.5 ± 1.1	49.2 ± 2.0	0.7 ± 0.1	0.9 ± 0.1
Isoschaftoside	12.0 ± 1.0	9.3 ± 0.7	73.6 ± 2.2	63.6 ± 2.7	7.9 ± 0.4	6.7 ± 0.1	9.1 ± 0.4	11.2 ± 0.1
Apigenin-6-C-glucosyl-8-C-arabinoside	2.4 ± 1.0	3.3 ± 1.1	13.7 ± 1.1	7.5 ± 1.2	14.5 ± 0.3	20.3 ± 0.3	-	-
Luteolin-6-C-glucoside	-	-	188.7 ± 0.8	139.5 ± 3.5	244.9 ± 3.7	397.8 ± 19.2	28.2 ± 6.3	22.1 ± 5.4
Apigenin 7-*O*-neohesperidoside	0.6 ± 0.3	0.6 ± 0.2	9.5 ± 0.3	11.9 ± 1.2	13.6 ± 0.1	38.3 ± 0.5	-	-
**(b)**								
Identified Compound	22 September 2020				14 October 2020		16 November 2020	
	CU7870		CU0618		CTC15	SP813250	RB966928	CTC4
	UHI		ULI		UHI	ULI	UHI	ULI
	5th H		3rd H		6th H	7th H	4th H	2nd H
Hydroxybenzoic acids	µg/g DW extract							
1-*O*-Vanilloyl-β-d-glucose	114.3 ± 14.5		11.4 ± 2.6		74.1 ± 0.7	1.2 ± 0.1	-	-
Vanillic acid	78.6 ± 4.1		4.2 ± 1.3		114.4 ± 5.5	2.5 ± 0.8	50.7 ± 2.3	8.8 ± 0.1
2,5-Dihydrobenzoic acid	10.0 ± 0.7		29.5 ± 1.7		53.6 ± 0.5	57.6 ± 0.8	19.9 ± 0.3	17.8 ± 0.3
Gentisic acid 2-*O*-β-glucoside	-		5.9 ± 0.5		-	2.6 ± 0.2	0.6 ± 0.0	-
Gentisic acid 5-*O*-β-glucoside	-		5.9 ± 0.7		-	2.6 ± 0.2	0.4 ± 0.3	-
4-Hydroxybenzoic acid	1.3 ± 0.1		18.2 ± 1.6		49.8 ± 0.2	26.7 ± 4.2	12.9 ± 0.2	11.5 ± 0.2
3,4-Dihydroxybenzaldehyde	-		-		1.7 ± 0.3	-	-	-
4-Hydroxybenzaldehyde	9.4 ± 1.0		9.6 ± 0.7		23.5 ± 0.9	24.5 ± 0.8	8.4 ± 0.1	8.2 ± 0.1
Hydroxycinnamic acids	µg/g DW extract							
Neochlorogenic acid	-		12.1 ± 0.0		28.5 ± 2.5	4.1 ± 0.8	-	-
Chlorogenic acid	3.2 ± 0.4		8.5 ± 7.1		42.9 ± 2.4	12.5 ± 0.3	3.2 ± 0.1	3.2 ± 0.1
4-Caffeoylquinic acid	-		8.8 ± 0.0		20.7 ± 1.2	3.9 ± 0.2	-	-
5-*O*-Feruloylquinic acid	4.9 ± 0.3		71.7 ± 2.9		149.6 ± 8.3	46.6 ± 5.7	17.5 ± 0.2	14.5 ± 0.1
*trans*-3-Feruloylquinic acid	4.4 ± 0.3		5.3 ± 0.7		48.1 ± 10.0	12.6 ± 1.0	10.4 ± 0.1	8.3 ± 0.0
Feruloylquinic acid isomer	-		46.5 ± 2.9		-	28.2 ± 1.1	-	-
Caffeic acid	2.1 ± 0.2		4.1 ± 0.2		50.3 ± 4.3	14.9 ± 0.8	4.3 ± 0.1	4.1 ± 0.0
Ferulic acid	18.3 ± 0.7		27.7 ± 0.5		20.7 ± 0.1	21.7 ± 0.4	-	-
*p*-Coumaric acid	37.2 ± 2.7		29.1 ± 0.4		40.8 ± 0.4	41.3 ± 0.9	20.5 ± 0.2	23.5 ± 0.2
Caffeoylquinic acid	1.8 ± 0.1		14.5 ± 0.5		23.2 ± 1.7	16.9 ± 1.4	6.3 ± 0.1	6.0 ± 0.0
4,5-Dicaffeoylquinic acid	-		4.9 ± 0.2		-	2.2 ± 0.2	-	-
Flavones	µg/g DW extract							
Apigenin-8-C-glucoside	10.7 ± 0.2		32.6 ± 0.6		49.3 ± 0.5	32.3 ± 0.5	8.3 ± 0.6	9.7 ± 0.1
Isovitexin 2″-O-arabinoside	1.5 ± 0.1		3.3 ± 0.1		-	-	-	-
Isoschaftoside	17.9 ± 1.0		37.7 ± 0.7		33.0 ± 0.2	33.4 ± 0.7	14.8 ± 0.2	17.5 ± 0.7
Apigenin-6-C-glucosyl-8-C-arabinoside	2.1 ± 0.1		3.9 ± 0.2		7.9 ± 0.5	11.4 ± 0.2	3.7 ± 0.0	4.1 ± 0.1
Luteolin-6-C-glucoside	19.9 ± 0.1		47.8 ± 1.1		213.6 ± 3.9	112.4 ± 2.9	24.3 ± 0.4	21.9 ± 1.5
Apigenin-7-O-neohesperidoside	0.8 ± 0.0		1.3 ± 0.1		7.5 ± 0.4	1.4 ± 0.1	-	-

**Table 4:** Antioxidant activity (ABTS and DPPH) measured in sugarcane straw extracts from plants collected in the Guariba and Valparaiso areas with borer high- and low-infection levels from plants harvested between June and November 2020 from different harvests (1st–7th).

Geographic Area	Harvesting Date	Variety	Borer Infection	Harvest	ABTS	DPPH
Bonfim	4 June 2020	CTC9001	High	1st	3.4 ± 0.0	8.8 ± 1.4
		CTC9001	Low	1st	2.3 ± 0.0	4.7 ± 0.0
	3 July 2020	CTC9001	High	1st	1.8 ± 0.1	5.3 ± 0.5
		RB966928	Low	7th	2.2 ± 1.5	6.8 ± 4.1
	24 July 2020	RB966928	High	1st	ND	ND
		RB966928	Low	1st	ND	ND
	8 August 2020	RB985476	High	1st	1.5 ± 0.1	1.4 ± 0.2
		CTC4	Low	3rd	1.5 ± 0.1	1.5 ± 0.3
	22 September 2020	CU7870	High	4th	1.2 ± 0.0	1.1 ± 0.0
		CU7870	Low	4th	1.5 ± 0.1	2.1 ± 0.0
	14 October 2020	RB985476	High	2nd	0.9 ± 0.0	1.0 ± 0.1
		SP803280	Low	4th	1.0 ± 0.0	1.2 ± 0.1
	16 November 2020	CU7870	High	1st	ND	ND
		CU7870	Low	1st	ND	ND
Univalem	4 June 2020	RB966928	High	1st	3.3 ± 0.0	6.9 ± 0.6
		CTC9001	Low	1st	3.6 ± 0.0	9.1 ± 1.1
	3 July 2020	CTC15	High	7th	1.4 ± 0.1	3.1 ± 1.0
		CTC15	Low	5th	1.7 ± 0.1	4.6 ± 0.2
	24 July 2020	CU7870	High	2nd	ND	ND
		CTC4	Low	1st	ND	ND
	8 August 2020	RB966928	High	3rd	2.9 ± 0.0	3.8 ± 0.2
		CU7870	Low	4th	2.9 ± 0.1	4.0 ± 0.5
	22 September 2020	CU7870	High	5th	1.2 ± 0.1	1.2 ± 0.2
		CU0618	Low	3rd	1.4 ± 0.0	1.4 ± 0.0
	14 October 2020	CTC15	High	6th	2.0 ± 0.0	5.0 ± 0.8
		CTC15	Low	5th	1.3 ± 0.1	2.4 ± 0.5
	16 November 2020	CU7870	High	2nd	ND	ND
		CTC4	Low	1st	ND	ND

ND, not determined.

**Table 5:** Summary of the effect of collection time (date) (X_1_), variety (X_2_), geographical area (X_3_), borer infection level (%) (X_4_), and harvest number (X_5_) on polyphenolic class content and antioxidant activity (ABTS and DPPH) obtained in extracts evaluated for sugarcane straw according to the factorial experimental design.

Estimated Effect of β	Hydroxybenzoic Acids	Hydroxycinnamic Acids	Flavones	ABTS	DPPH
Intercept	157.15 **	57.81	214.59 ***	2.78 ***	0.57
Collection time (X_1_)	−279.32	1463.86	100.54	5.57	−11.86
Variety (X_2_)	472.95 ***	−1147.95	27.59	−10.31 *	10.23
Geographical area (X_3_)	−175.01 **	460.15	−59.15	1.49	−4.46
Borer infection level (X_4_)	−281.58 *	−678.45	−64.99	1.90	1.94
Harvest number (X_5_)	−288.93 *	−1549.26 **	−74.35	0.02	6.15
X^2^_1_	−103.11	−5.56	−158.66	−4.16 *	6.25
X^2^_2_	830.57 ***	−160.05	−190.47	−15.60 *	21.44
X^2^_3_	-	-	-	-	-
X^2^_4_	−169.99	−196.20	21.38	0.37	8.80
X^2^_5_	375.45 ***	1625.61 ***	316.39 ***	0.07	−2.74
X_1_ X_2_	−1070.49 ***	−488.82	−249.28	14.78 *	−18.51
X_1_ X_3_	−392.48 ***	−1681.32 ***	−109.58	0.77	0.39
X_1_ X_4_	−313.79 *	1316.06	116.41	−0.80	−3.40
X_1_ X_5_	44.96	543.74	277.76 ***	5.15 **	−4.00
X_2_ X_3_	224.98 **	1310.92 ***	27.71	−2.11 **	−1.94
X_2_ X_4_	236.49	−1483.73 *	−141.22	1.49	0.44
X_2_ X_5_	−443.79 ***	−2028.47 ***	−346.02 *	−4.53	5.55
X_3_ X_4_	−103.02	274.62	24.52	1.84	−5.49
X_3_ X_5_	217.70 ***	593.82 *	167.65*	−0.38	−4.47
X_4_ X_5_	101.01	−486.24	7.49	−1.42	5.48
R^2^	0.949	0.810	0.857	0.99	0.92
R^2^ *Adjusted*	0.922	0.710	0.781	0.84	0.84
*RMSE*	7.455	38.418	8.948	0.04	0.12

* *p* ≤ 0.05; ** *p* ≤ 0.01; *** *p* ≤ 0.001; beta (β) symbols represent unstandardized coefficients.

**Table 6:** Prediction values (±confidence intervals at a 95% confidence level) and desirability for the optimal content of polyphenol classes (hydroxybenzoic acids, hydroxycinnamic acids, and flavones, (µg/g dry extract) for the best harvesting conditions considering collection date, variety, geographic area, borer infection level and harvest number according to the central composite design (CCD).

Parameter	Optimum Harvesting Conditions for All the Parameters Combined	Predicted Values at Optimum Harvesting Conditions	Desirability
Hydroxybenzoic acids	Collection date: October 2020 Variety: CU 0618 Geographic area: Guariba Infection level: 13.81% Harvesting: 7th	977.59 ± 482.52	1.0
Hydroxycinnamic acids		1336.16 ± 764.92	
Flavones		1660.49 ± 388.94	
ABTS		4.84 ± 5.92	
DPPH		9.96 ± 17.95	

**Table 7:** The optimal multilayer perceptron (MLP) networks developed for single-variable outputs.

Target Output	Optimal Neural Network	Correlation Coefficients		
		Training Data	Testing Data	Validation Data
Hydroxybenzoic acids	MLP 20-5-5	0.994	0.956	0.993
	MLP 20-3-5	0.978	0.935	0.994
	MLP 20-4-5	0.919	0.929	0.892
	MLP 20-4-5	0.981	0.915	0.975
	MLP 20-3-5	0.966	0.933	0.958
Hydroxycinnamic acids	MLP 20-5-5	0.991	0.980	0.956
	MLP 20-3-5	0.940	0.954	0.880
	MLP 20-4-5	0.895	0.931	0.864
	MLP 20-4-5	0.894	0.974	0.984
	MLP 20-3-5	0.949	0.956	0.837
Flavones	MLP 20-5-5	0.989	0.992	0.973
	MLP 20-3-5	0.950	0.973	0.967
	MLP 20-4-5	0.888	0.834	0.886
	MLP 20-4-5	0.976	0.940	0.989
	MLP 20-3-5	0.904	0.951	0.928
ABTS	MLP 20-5-5	0.997	0.936	0.563
	MLP 20-3-5	0.983	0.990	0.764
	MLP 20-4-5	0.770	0.931	0.948
	MLP 20-4-5	0.956	0.968	0.828
	MLP 20-3-5	0.898	0.969	0.838
DPPH	MLP 20-5-5	0.979	0.985	0.742
	MLP 20-3-5	0.941	0.967	0.621
	MLP 20-4-5	0.868	0.910	0.862
	MLP 20-4-5	0.955	0.972	0.702
	MLP 20-3-5	0.911	0.964	0.781

**Table 8:** Comparison of optimization and prediction capabilities of response surface methodology (RSM) and artificial neuron network (ANN) for the extraction of phenolic compounds, organized by class, and for antioxidant activity (ABTS and DPPH) from sugarcane straw harvested under different biotic and abiotic conditions.

Response	Modeling Method	Optimal Neural Network	R^2^	RMSE
Hydroxybenzoic acids	RSM		0.949	7.455
	ANN	MLP 20-5-5	0.989	4.461
		MLP 20-3-5	0.956	4.356
		MLP 20-4-5	0.85	4.734
		MLP 20-4-5	0.963	5.449
		MLP 20-3-5	0.933	4.213
Hydroxycinnamic acids	RSM		0.810	38.418
	ANN	MLP 20-5-5	0.983	7.869
		MLP 20-3-5	0.885	18.339
		MLP 20-4-5	0.801	16.526
		MLP 20-4-5	0.799	17.425
		MLP 20-3-5	0.901	19.013
Flavones	RSM		0.857	8.948
	ANN	MLP 20-5-5	0.978	3.908
		MLP 20-3-5	0.902	4.020
		MLP 20-4-5	0.789	5.154
		MLP 20-4-5	0.952	4.928
		MLP 20-3-5	0.817	5.839
ABTS	RSM		0.990	0.039
	ANN	MLP 20-5-5	0.994	0.011
		MLP 20-3-5	0.966	0.024
		MLP 20-4-5	0.593	0.087
		MLP 20-4-5	0.913	0.083
		MLP 20-3-5	0.807	0.048
DPPH	RSM		0.920	0.118
	ANN	MLP 20-5-5	0.959	0.089
		MLP 20-3-5	0.886	0.119
		MLP 20-4-5	0.753	0.207
		MLP 20-4-5	0.913	0.220
		MLP 20-3-5	0.829	0.144

## Data Availability

Data is contained within the article.
